# Discontiguous or Contiguous Spread Patterns Affect the Functional Staging in Patients With Sporadic Amyotrophic Lateral Sclerosis

**DOI:** 10.3389/fneur.2019.00523

**Published:** 2019-05-22

**Authors:** Li Zhenfei, Duan Shiru, Zhou Xiaomeng, Cao Cuifang, Liu Yaling

**Affiliations:** ^1^Department of Neurology, The Second Hospital of Hebei Medical University, Shijiazhuang, China; ^2^Key Laboratory of Hebei Neurology, Shijiazhuang, China

**Keywords:** Amyotrophic lateral sclerosis, prion-like mechanism, electrophysiology, spread pattern, functional staging

## Abstract

**Objective:** This study aimed to investigate whether the spread pattern affects functional staging in amyotrophic lateral sclerosis (ALS). We examined the spreading patterns of disease following symptom onset and the affected regions in ALS using electromyography.

**Methods:** This study reviewed the medical records of 103 patients with sporadic ALS in the Second Hospital of Hebei Medical University from 2012 to 2017. According to the clinical manifestation and the distribution of the affected regions on electromyography, spread patterns were classified as discontiguous or contiguous. The patients were graded according to the ALS-Milano-Torino staging (MITOS) system.

**Results:** The clinical spread patterns were contiguous in 91.5% of patients and discontiguous in 8.5% of patients. The electrophysiological spread patterns were contiguous in 87.4% of patients and discontiguous in 12.6% of patients. Sex, age, or delay in diagnosis did not affect the clinical or electrophysiological spread patterns. No significant correlation was observed between the clinical classification and the ALS-MITOS grade, but the electrophysiological spread was significantly correlated with the ALS-MITOS.

**Conclusion:** This study provides evidence that not all ALS patients show contiguous clinical or electrophysiological spread patterns. The electrophysiological spread pattern can affect the functional staging in ALS patients.

## Introduction

Amyotrophic lateral sclerosis (ALS) is a fatal degenerative disease affecting the motor nervous system. There are several hypotheses about the pathogenesis of ALS. Prion-like protein aggregation has recently been proposed as a new mechanism of ALS. Prion Protein (PrP) is a type of self-proliferating and contagious cell membrane protein and is widely expressed in the body, especially in the nerve tissues (1). The prion-like mechanism is considered to involve a misfolded protein that is similar to PrP and can reproduce and thus accumulate. The prion-like mechanism is one of the pathophysiological mechanisms of neurodegenerative disease. This mechanism appears not only in ALS but also in other neurodegenerative diseases, such as Parkinson's disease (2) and Alzheimer's disease (3). The motor neurons in ALS have misfolded oligomers or protein inclusions composed of TAR DNA-binding protein 43 (TDP-43), fused in sarcoma (FUS), or Cu-Zn superoxide dismutase 1 (Cu-Zn SOD1). Some studies with cell and animal models have shown that misfolded SOD1 and TDP-43 could propagate and be transmitted to neighboring neuronal cells (4, 5).

Clinically, the major pathological changes of ALS occur in four regions: the bulbar, cervical spinal cord, thoracic spinal cord, and lumbar spinal cord; the disorder is further classified based on the affected region. According to the prion-like mechanism, ALS is a disease with a focal onset and a contiguous spread. However, the compensatory predominance of motor neurons can mask the clinical signs.

When more than 1/3 of the lower motor neurons (LMNs) are lost, corresponding clinical symptoms begin to appear (6). Electromyography can partially detect these clinical symptoms. Even at the pre-symptomatic stage of ALS, electromyogram is sufficiently sensitive for evaluating changes in each segment of the LMNs. Needle electromyogram is a powerful method to assess whether the lesion involves the adjacent segments of the LMNs. In this study, needle electromyogram was used to investigate the spread of disease along the adjacent segments of LMNs.

The ALS-Milano-Torino staging (MITOS) system has been proposed as a new tool for examining the progress of ALS (7). The ALS-MITOS system uses the ALS functional rating scale revised (ALSFRS-R) to evaluate four functional domains, and functional staging can be accurately performed according to the loss of function, which is consistent with the progression of the disease. In addition, the ALS-MITOS system overcomes the shortcomings of the nonlinear and multidimensional characteristics of the ALSFRS-R (8). Moreover, the ALS-MITOS system can be used to evaluate the quality of life of patients and the cost of health services.

## Materials and Methods

A retrospective analysis was performed using the complete medical history, examination records, and electromyogram data of 103 sporadic ALS patients at the Neurology Outpatient or Inpatient Department in the Second Hospital of Hebei Medical University from May 2012 to October 2017. Volunteers participated in the study after giving informed consent, which was approved by our hospital. All patients were diagnosed by a neurologist at the Second Hospital of Hebei Medical University. They were all diagnosed with probable, laboratory-supported, or definite ALS according to the El Escorial revised criteria. Patients with a family history of primary lateral sclerosis; clinically suspected ALS or frontotemporal dementia; or severe heart, lung, liver, or kidney diseases were excluded from this study. The general information of all patients, the length of diagnosis delay, the initial location, and the complete electromyography data were collected. The initial location was determined according to the patient's history and physical examination, and the secondary location was determined according to the clinical manifestations.

Cases involving early symptoms of dysphagia, dysarthria, tongue muscle atrophy and fibrillation, and reduced or absent soft palate and (or) pharyngeal reflex were classified as the bulbar type of ALS. Cases involving early symptoms of upper limb muscle atrophy, weakness, fasciculation, and reduced or absent tricep and bicep reflexes were classified as the cervical spinal cord type of ALS. Cases involving early symptoms of lower limb muscle atrophy, weakness, fascicular tremors, and/or reduced or absent knee-jerk reflex were classified as the lumbar spinal cord type of ALS.

This study considered seven spread patterns from onset to diagnosis according to the order of the affected regions: superior interposed, middle interposed, inferior interposed, circular, crossed, rostrocaudal, and caudorostral regions (9). Depending on whether the regions affected by ALS are contiguous, the affected regions could be divided into contiguous and discontiguous types. Contiguous types included rostrocaudal, caudorostral, circular, and middle-interposed types. Discontiguous types included superior interposed, inferior interposed, and cross types.

One or two muscles in each region of the lower motor neuron were observed by needle electromyogram. If there was a fibrillation potential, positive sharp wave, fasciculation and/or high amplitude, and wide time movement unit potential in the muscle, the corresponding region was considered affected. All electromyogram tests were conducted by an experienced neurologist who had at least 5 years of professional electromyogram experience.

According to the distribution of the location on electromyogram, the extent of involvement was divided into two types: contiguous and discontiguous. The contiguous type included (1) the total involvement of all four segments on electromyography and (2) the involvement of the two or three segments of the adjacent body regions. For the discontiguous type, the affected patient's segments shown on electromyogram were not present in adjacent segments.

The ALS-MITOS system is based on the assessment of four functional domains measured by the ALSFRS-R. The ALS-MITOS system could reliably identify relevant stages of disease in patients according to the number of lost functions. Five stages were defined: stage 0 indicates functional involvement and no loss of function in any domain; stages 1–4 represent the number of domains in which function is lost; and stage 5 indicates death. The MITOS is a good way of measuring quality of life (10).

Results were analyzed using the Statistical Package for Social Sciences (version 22.0; SPSS, Shanghai, China). Pearson's chi-square test or independent samples *t*-test was used to examine the differences between different variables. Skewed values were log-transformed before analysis. A *p*-value of < 0.05 was considered statistically significant.

## Results

A total of 103 patients (60 males [58.3%] and 43 females [41.7%]) were included in this study. The ratio of men to women was 1.36:1. The mean time from onset to diagnosis was 12.60 ± 10.34 months (range, 0.6–60 months). The average age of onset was 54.31 ± 9.68 years (range, 27–72 years). There were seven patients (6.8%) with age of onset <40 years; 61 patients (59.2%) were older than 40 years old and younger than 60 years old; 35 patients (34%) were above 60 years old. The diagnosis delay time in 45 patients (43.7%) was <12 months, and that in 58 patients (56.3%) was 12 months or more.

Demographic and clinical characteristics of patients with the bulbar, cervical spinal cord, and lumbar spinal cord types of ALS were analyzed in this study (Table 1). The initial region affected in ALS was associated with sex. Most of the patients with disease onset in the cervical spinal cord were male (male: female, 2.05), and more female patients had disease onset in the bulbar (male: female 0.53). The proportions of men and women with the lumbar spinal cord disease were approximately equal (male: female, 1.2). In addition, the initial region affected in ALS was also related to age in this case series. Disease onset in the cervical spinal cord or lumbar spinal cord was more common among the patients aged 40–60 years. Most patients with the disease onset in the bulbar were more than 60 years old. Among patients with disease onset in the cervical spinal cord, the numbers of patients with a <12-month and >12-month delay in diagnosis were equal. Among patients with disease onset in the bulbar and lumbar spinal cord, the majority were diagnosed after a delay time of more than 12 months, but no significant differences were observed in the numbers diagnosed within and after 12 months.

**Table 1:** Demographic and clinical characteristics of patients according to regions of onset.

	**Bulbar**	**Cervical cord**	**Lumbar cord**	***P***
**Gender**				
Male	7	41	12	
Female	13	20	10	0.037
**Age at diagnosis (years)**				
<40 (y)	0	3	4	
≥40 & <60 (y)	9	39	13	
≥60 (y)	11	19	5	0.035
**Delay time to diagnosis (months)**				
<12	8	31	6	
≥12	12	30	16	0.151
				

In eight patients in total, clinical manifestation did not progress over time and were therefore not included in the analysis. Among the remaining 95 patients, 87 patients (91.5%) were classified as the contiguous type, and eight (8.5%) the discontiguous type. There were no differences in the progression of clinical manifestation, gender, age, or delay in diagnosis between discontiguous and contiguous types (Table 2). In addition, 90 patients (87.4%) had the electromyogram contiguous type, and 13 patients (12.6%) the discontiguous type. No significant differences were noted in patient sex, age, or course of disease between the electromyogram types (Table 2).

**Table 2:** Demographic and clinical characteristics of patients according to spread patterns.

	**Clinical spread continuous discontiguous**		***P***	**Electrophysiological spread continuous discontiguous**		***P***
**Gender**						
Male	52	4		52	8	0.797
Female	35	4	0.713	38	5	
**Age at diagnosis (years)**						
<40 (y)	5	2		6	1	0.915
≥40 & <60 (y)	50	5		54	7	
≥60 (y)	32	1	0.084	30	5	
**Delay time to diagnosis (months)**						
<12	39	3		40	5	0.684
≥12	48	5	0.494	50	8	

The type of clinical spread was significantly related to the location of onset. Cases with disease onset in the cervical spinal cord were more likely to progress to become those with the contiguous spread. However, no significant correlation was observed between the type of electromyographic spread and the location of onset (Table 3).

**Table 3:** Correlation between spread patterns and regions of onset.

	**Onset regions**			
	**Bulbar**	**Cervical cord**	**Lumbar cord**	***P***
**Clinical spread**				
Continuous	17	53	17	0.003
Discontinuous	3	0	5	
**Electromyographic spread**				
Continuous	16	52	22	0.110
Discontinuous	4	9	0	

Based on the ALS-MITOS staging, there were 35 patients (33.9%) at stage 0, 35 patients (33.9%) at stage 1, 19 patients (18.5%) at stage 2, 11 patients (10.7%) at stage 3, and three patients (2.9%) at stage 4. No patients were observed at stages 5. No significant correlation was found between the clinical classification and ALS staging (Table 4), but electromyographic classification was significantly correlated with ALS staging (Table 4).

**Table 4:** Correlation between spread patterns and the amyotrophic lateral sclerosis-Milano-Torino staging (ALS-MITOS).

		**ALS-MITOS**				
	**Stage 0**	**Stage 1**	**Stage 2**	**Stage 3**	**Stage 4**	***P***
**Clinical spread**						
Continuous	12	30	21	20	4	0.144
Discontinuous	2	1	2	1	2	
**Electromyographic spread**						
Continuous	14	31	21	20	4	0.005
Discontinuous	6	3	2	1	1	

## Discussion

The actual location of onset of ALS is debated. Irrespective of the ALS onset zone, the clinical manifestations seem to start at one site and then spread to other regions over time in the majority of patients (11–15).

Disease onset in the cervical spinal cord is the most common among ALS patients, as demonstrated in previous studies (12, 13). An autopsy study indicated that the degree of motor neuron damage was different in different segments (16). Another autopsy study demonstrated that a more pronounced loss is found in the C8 anterior horn cells that dominate in the distal hand muscles than in the C6 anterior horn cells that dominate in the upper arm muscles (17). Motor neurons with longer axons (15, 18), larger motor neurons (17), and fatigue-prone motor neurons (19) are thought to be more vulnerable to ALS.

The most patients were between 40 and 60 years of age (Table 1). The location of onset was also related to age. The onset of lumbar spinal cord disease was more common in patients younger than 40 years old, while the onset of bulbar oblongata was more common in patients older than 60 years old. Patients with the cervical spinal cord disease were more common in each age group.

Patients in this study were classified according to the clinical spread patterns of disease. The clinical manifestations were correlated with the site of onset, for example, bulbar disease correlates with brainstem involvement, cervical cord involvement with arm involvement and the lumbar far cord with leg involvement. The involvement of the thoracic cord is often hidden because of the lack of obvious symptoms and signs; thus, the clinical manifestations do not include the symptoms and signs of the affected thoracic cord. If the primary and secondary sites are adjacent horizontally or vertically, the affected regions are classified as contiguous; otherwise, they are defined as discontiguous. The electrophysiological examination can be used to examine the clinical manifestations and detect early denervation damage in the thoracic spinal cord. According to whether the damage in the four regions, i.e., the bulbar oblongata, cervical spinal cord, thoracic spinal cord, and lumbar spinal cord was contiguous, cases were divided into contiguous and discontiguous types. It was found that both clinical spread and electrophysiological spread were mainly contiguous. This contiguous spread is consistent with the characteristics of prion-like diffusion between misfolded proteins in adjacent cells. However, a small number of clinical and electrophysiological spreads do not follow this rule and show discontiguous progression. In an electrophysiological study of ALS, T10-L5 muscle involvement was contiguously observed. It was found that 14 out of 36 cases were of the discontiguous type. In patients with the discontiguous type, damaged LMNs in the lumbosacral region spread in a horizontal or radial manner (20). However, regardless of the direction of spread, most of LMNs spread along adjacent bodies. Therefore, the theory of focal onset and simple spreading along adjacent sites cannot fully explain the development of ALS, and multifocal disease and local spreading may be implicated in this development. In our study, the number of discontiguous types is varied according to the clinical or electrophysiological methods adopted. In the clinical setting, few discontiguous types (only 8 patients) were observed. The number of discontiguous types increased in electrophysiological typing. Collectively, the above results suggest that more patients of the discontiguous type might have been identified if we examined more muscles or more segments of the spinal cord. We did not find any differences in clinical features, such as age, sex, and delayed diagnosis, between contiguous and discontiguous electrophysiological patients. However, if more accurate methods were used, more patients with the discontiguous type could have been identified. It is possible to find clinical, pathophysiological, and genetic differences in patients with the discontiguous type compared with those with the contiguous type. This is the direction of our future work.

The ALS-FRS system assesses functions in four key regions, including swallowing, walking, self-care, communication, and breathing. ALS-MITOS stages are determined according to ALS-FRS results. The ALS-FRS system can partly reflect the functional state of ALS patients. This study showed no significant relationship between the clinical progression pattern and the ALS grade. The electrophysiological progression pattern was significantly correlated with the functional stage of ALS. Patients in the electrophysiological discontiguous group had more functions than those in the contiguous group, and patients in the electrophysiological contiguous group were more severe than those in the discontiguous group.

Some studies have the opposite point of view. In a study on the effects of different progression patterns on survival, the prognosis of patients with the intermediate insertion (discontinuous) progression patterns was worse, while the prognosis of patients with the caudorostral type was better than that with other progression patterns (9). This may be because the distance from the lumber spinal Cord to the bulbar affecting respiration and swallowing function is the farthest. Conversely, the discontiguous onset of the bulbar symptoms or progression from the lumbar spine to the bulbar due to the early involvement of the bulb is more likely to affect early respiratory and swallowing function. This may be one of the causes of poor prognosis of patients with discontiguous progression. The present results may be related to the different evaluation methods. In 13 patients with electrophysiological discontiguous progression, 12 had no abnormal spontaneous potential in the muscles dominating in the thoracic cord. The respiratory muscles dominating in the thoracic segments were temporarily unaffected in almost all patients with the discontiguous type. This may be because the ALS-MITOS system reflects earlier discontiguous staging and less clinical manifestations.

Because upper motor neuron (UMN) signs are less discernible symptoms for ALS patients, our study only investigated the involvement of LMNs. Another limitation of our study is its retrospective design. Further prospective studies with detailed information on UMN, LMN behavior, clinical electrophysiology, and regional patterns of spread will be required to validate and confirm our conclusions.

## Ethics Statement

This study was carried out in accordance with the recommendations of spread patterns affect the functional staging in patients with sporadic amyotrophic lateral sclerosis, The Second Hospital of Hebei Medical University committee' with written informed consent from all subjects. All subjects gave written informed consent in accordance with the Declaration of Helsinki. The protocol was approved by the Second Hospital of Hebei Medical University committee.

## Author Contributions

All authors listed have made a substantial, direct and intellectual contribution to the work, and approved it for publication.

### Conflict of Interest Statement

The authors declare that the research was conducted in the absence of any commercial or financial relationships that could be construed as a potential conflict of interest.
